# Mild Autonomous Cortisol Secretion in Congenital Adrenal Hyperplasia Managed With Mini Back Scope Adrenalectomy

**DOI:** 10.1210/jcemcr/luaf295

**Published:** 2025-12-18

**Authors:** Tobias Carling, Raisa A Mayers, Meredith LaRue, Alejandra Kalik

**Affiliations:** Carling Adrenal Center, Tampa, FL 33615, USA; Department of Surgery, Hospital for Endocrine Surgery, Tampa, FL 33615, USA; South Florida Endocrine Center, Palm Beach Gardens, FL 33410, USA; Department of Surgery, Hospital for Endocrine Surgery, Tampa, FL 33615, USA; Department of Pathology, Hospital for Endocrine Surgery, Tampa, FL 33615, USA

**Keywords:** adrenal, congenital adrenal hyperplasia, mild autonomous cortisol secretion, adenoma, surgery

## Abstract

A 55-year-old woman, diagnosed with 21-hydroxylase deficiency congenital adrenal hyperplasia (CAH) at age 7 years in the mid-Atlantic United States and living mostly outside the country, recently developed mild autonomous cortisol secretion (MACS) with bilateral adrenal lesions. Initially managed with 20 years of glucocorticoid therapy for hyperandrogenism, she discontinued treatment at age 33 due to iatrogenic Cushing syndrome features, remaining off therapy for close to 20 years with stable eucortisolemia. Recent weight gain prompted evaluation, revealing elevated cortisol (3.1 µg/dL postdexamethasone suppression; SI: 86.1 nmol/L), undetectable ACTH, and a dominant right adrenal tumor (5.1 cm) but with bilateral lesions. She underwent right mini back scope adrenalectomy (MBSA). The pathology confirmed a benign adenoma, and postoperative hypercortisolemia resolved, with transient postoperative hypocortisolism managed with glucocorticoid replacement. This case highlights the rare emergence of MACS in people with longstanding CAH, underscoring the need for lifelong adrenal monitoring and the effectiveness of MBSA.

## Introduction

Congenital adrenal hyperplasia (CAH) stems from inherited enzyme deficiencies that impair cortisol synthesis, with 21-hydroxylase deficiency accounting for 95% to 99% of cases, caused by mutations in the *CYP21A2* gene [1, 2]. Deficiency in 21-hydroxylase impairs cortisol production, leading to adrenal insufficiency, often necessitating glucocorticoid therapy [3]. Prolonged use can trigger iatrogenic Cushing syndrome [4], while endogenous hypercortisolism, such as mild autonomous cortisol secretion (MACS) from an adrenal adenoma, remains a rare complication. Adrenal (primary) hypercortisolism (AHC) is the most common cause of endogenous hypercortisolism. Like other endocrine tumor manifestations involving hormone excess, AHC spans a biochemical spectrum ranging from mild (MACS) to severe (overt Cushing syndrome). This spectrum correlates imperfectly with clinical severity, adrenal tumor cell mass, or efficiency of hormone hypersecretion per tumor cell. AHC's terms, including Cushing syndrome, subclinical Cushing syndrome, and MACS, are often used interchangeably, which can create clinical and scientific challenges. Recent research by Sahlander et al has shown a significantly elevated prevalence of CAH among individuals with adrenal tumors, hinting at chronic ACTH overstimulation as a potential driver [5]. This case illustrates the unexpected development of an adrenal adenoma and MACS decades after a CAH diagnosis, emphasizing the critical need for ongoing adrenal surveillance in this population.

## Case Presentation

A 55-year-old woman with type 2 diabetes and hypothyroidism sought care for recent weight gain and mild features of MACS. Her medical history began at age 6, approximately 50 years ago, when she was diagnosed with nonclassical 21-hydroxylase deficiency CAH in the mid-Atlantic United States, and although the details of her pediatric endocrinology workup are not available, she was presumed to have a nonclassical phenotype. She self-reports that she had early-onset puberty with pubic hair, hirsutism, and acne by the time she was 6 or 7 years old. Her older sister shared a similar diagnosis but with an earlier onset (newborn, although the medical records are unavailable). Having lived mostly outside the United States, she was prescribed glucocorticoids (initially prednisone but later transitioned over to hydrocortisone) for 20 years to manage symptoms of hirsutism, acne, and menstrual irregularity. At age 33, iatrogenic Cushing syndrome manifested as moon facies and truncal obesity, prompting her to discontinue glucocorticoid therapy herself. For the next 20 years, she maintained clinical and biochemical eucortisolemia, confirmed by normal cortisol levels. However, over the past 5 years, she experienced weight gain (from a weight of 165 lbs.; 78.4 kg to 210 lbs.; 95.3 kg) now with a body mass index of 36.4 kg/m², muscle weakness, decreased libido, neurocognitive decline, easy bruising, skin thinning, mild hirsutism, and alopecia, signaling a new phase of hypercortisolism.

## Diagnostic Assessment

**Figure 1.**
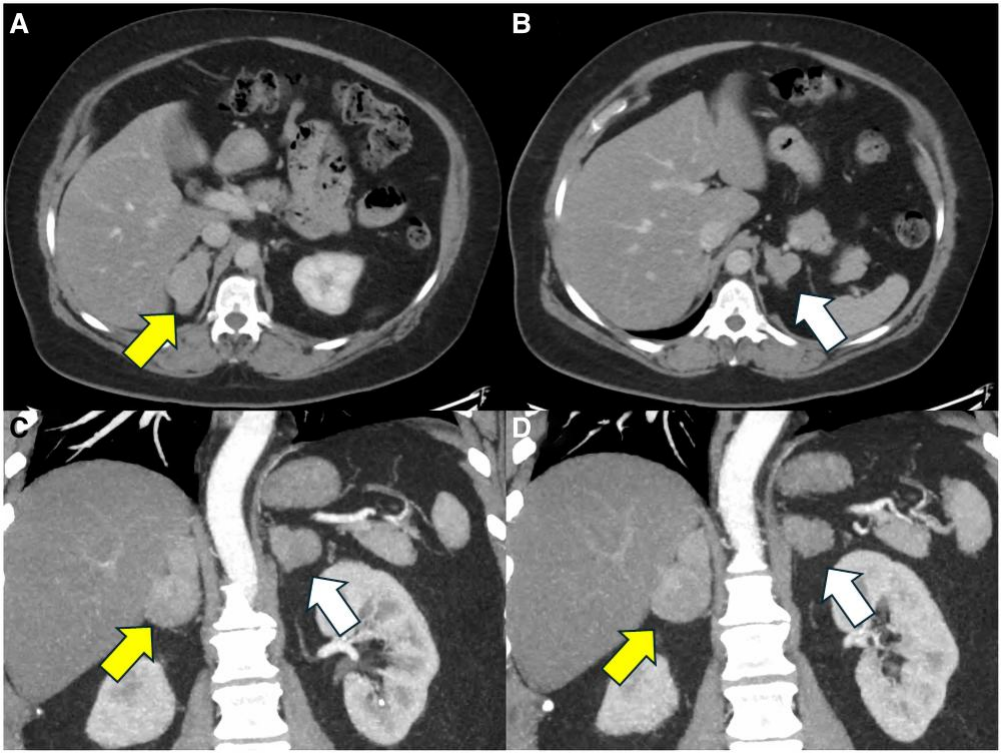
Representative CT scan images from the patient with MACS in the setting of CAH. CT scan of the abdomen showing bilateral adrenal lesions, with the right (yellow arrow) and left (white arrow) adrenal lesions, on axial (A and B) and coronal (C and D) views. All CT scan images depicted are in venous phase (60 seconds following contrast injection) according to a modified adrenal protocol of the Carling Adrenal Center and Hospital for Endocrine Surgery. The dominant right adrenal mass (5.1 cm, 21.5 Hounsfield units) and a left mass (2.7 cm, 11.5 Hounsfield units), indicating adenomas contributing to the recent development of MACS. Abbreviations: CAH, congenital adrenal hyperplasia; CT, computed tomography; MACS, mild autonomous cortisol secretion.

Given the symptoms, the patient was evaluated for suspected hypercortisolemia. An initial 1 mg overnight dexamethasone suppression test revealed a morning cortisol level of 3.1 µg/dL (86.1 nmol/L; normal <1.8 µg/dL; < 50 nmol/L), which when repeated was 2.4 µg/dL (66.7 nmol/L). ACTH levels were undetectable (repeated multiple times), and dehydroepiandrosterone sulfate (DHEA-S) was low at 63 µg/dL (reference range female ages 50-59: 15-170 µg/dL or 0.4-4.6 µmol/L). A 24-hour urine collection for free cortisol (25.3 µg, with upper normal reference range of 45 µg) and late-night salivary cortisol tests (repeated 4 times and ranging between 0.021 and 0.047 µg/dL with a reference range of 0.010-0.090 µg/dL) returned normal results, consistent with mild AHC, as opposed to overt Cushing syndrome. Genetic testing for nonclassical 21-hydroxylase deficiency CAH was recommended but declined, although based on the personal and family history, it is assumed that a mutation in the *CYP21A2* gene would be most consistent with the phenotype. Additional biochemical indices of the patient prior to adrenalectomy are summarized in Table 1. A computed tomography scan of the abdomen revealed bilateral adrenal masses, with the largest measuring 5.1 cm on the right adrenal gland and 2.7 cm on the left with noncontrast attenuation of 21.5 and 11.5 Hounsfield units, respectively. (Figure 1).

**Table 1.** Summary of the patient's biochemical indices relevant to nonclassical congenital adrenal hyperplasia and mild autonomous cortisol secretion at selected time points before and after right MBSA with corresponding postoperative hydrocortisone replacement dose

Serum labs (reference range)	Before right MBSA (2023-2024)	1-2 months after right MBSA	3-9 months after right MBSA	10-15 months after right MBSA
Hydrocortisone dose (daily)	Off therapy >2 decades	7.5-15 mg	7.5-10 mg	7.5 mg
17-Hydroxyprogesterone (35-200 ng/dL; 1.06-6.06 nmol/L)	Not available	—	394-2518 ng/dL (11.9-76.04 nmol/L)	477-1655 ng/dL (14.4-50.0 nmol/L); ACTH stim 60 minutes: 7730 ng/dL*^a^* (233.5 nmol/L)
ACTH (7-50 pg/mL; 1.54-11.0 pmol/L)	<5 pg/mL*^b^* (<1.1 pmol/L)	14.1-23.6 pg/mL (3.1-5.2 pmol/L)	11.8-18.9 pg/mL (2.6-4.2 pmol/L)	—
Cortisol, morning (5-25 µg/dL; 138-690 nmol/L)	18.2 µg/dL (502 nmol/L)	5.3-11 µg/dL (146-303 nmol/L)	2.1-12.3 µg/dL (58-339 nmol/L)	6.8-7.6 µg/dL (188-210 nmol/L)
Cortisol post-1 mg DST (<1.8 µg/dL; < 50 nmol/L)	2.4-3.1 µg/dL*^c^* (66-86 nmol/L)	—	—	—
Cosyntropin stimulated peak cortisol (>18 µg/dL; > 496 nmol/L)	—	10.2 µg/dL (281 nmol/L)	12.6 µg/dL (348 nmol/L)	—
Testosterone, total (15-70 ng/dL; 0.52-2.43 nmol/L)	42 ng/dL (1.46 nmol/L)	—	—	—
Free testosterone (0.3-1.9 ng/dL; 10-66 pmol/L)	1.2 ng/dL (42 pmol/L)	—	—	—
Estradiol (<5-54 pg/mL; < 18-198 pmol/L)	<5 pg/mL (<18 pmol/L)	—	6 pg/mL (22 pmol/L)	5 pg/mL (18 pmol/L)
DHEA-S (35-430 µg/dL; 0.95-11.6 µmol/L)	63 µg/dL (2.41 µmol/L)	—	92 µg/dL (2.49 µmol/L)	—
Androstenedione (30-200 ng/dL; 1.05-7.0 nmol/L)	156 ng/dL (5.45 nmol/L)	—	—	—
Progesterone (≤0.3 ng/mL follicular; ≤ 0.9 nmol/L)	0.2 ng/mL (0.6 nmol/L)	—	<0.1-0.2 ng/mL (<0.3-0.6 nmol/L)	—
21-Hydroxylase antibody	Negative	—	Negative	—

Units are in conventional and SI units.

Abbreviations: DHEA, dehydroepiandrosterone; DST, dexamethasone suppression test; MBSA, mini back scope adrenalectomy.

^*a*^ACTH stimulation test: baseline 944 ng/dL → 60 minutes 7730 ng/dL (confirmed by dilution); diagnostic of persistent nonclassical congenital adrenal hyperplasia.

^*b*^Numerous undetectable ACTH with elevated post-DST cortisol confirmed mild autonomous cortisol secretion.

^*c*^Repeated 1-mg dexamethasone suppression test several times. All postoperative laboratory studies were obtained on physiologic hydrocortisone replacement (7.5-15 mg daily).

## Treatment

**Figure 2.**
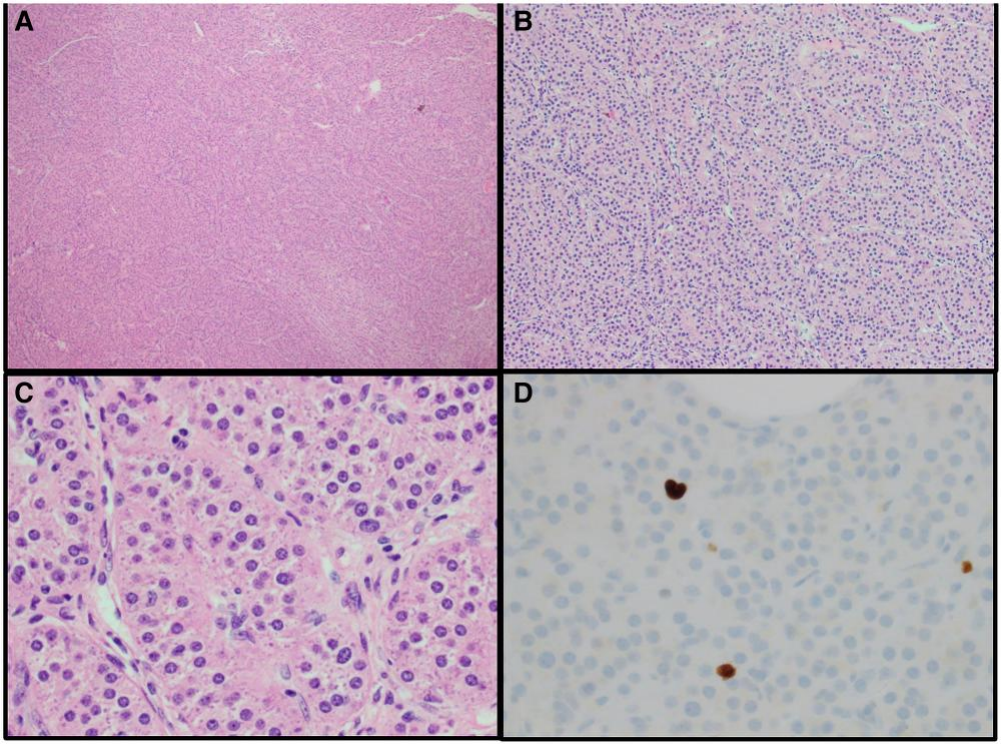
Histopathology of the resected right adrenal gland. Representative images from surgical pathology showing a 7.0 × 5.1 × 3.0 cm brown-orange lobulated adenoma with Weiss score 0 and Ki-67 2% to 3%, confirming benign features. Staining used hematoxylin and eosin (A-C) as well as Ki-67 proliferation marker immunostaining staining (D), using magnification of 40× (A), 100× (B), and 400× (C and D). The pathological diagnosis was consistent with an adenoma with a Weiss score of 0, and Ki-67 staining demonstrating a low proliferative rate of 2% to 3%, indicating benign features.

The patient underwent a right minimally invasive adrenalectomy using the mini back scope approach (MBSA; also known as posterior retroperitoneoscopic adrenalectomy). The rationale for resecting the right side first was related to its greater size and anticipated enhanced contribution to the hypercortisolism. In addition, the left adrenal gland on its inferior portion had the most normal adjacent adrenal tissue, which could be preserved during a future function-preserving (partial) MBSA should the patient need it following unilateral right adrenalectomy. The surgical procedure lasted 23 minutes and was completed without complications. Pathological examination of the excised adrenal gland revealed a 7.0 × 5.1 × 3.0 cm brown-orange, lobulated mass, consistent with an adenoma with a Weiss score of 0, and Ki-67 staining demonstrating a low proliferative rate of 2% to 3%, indicating benign features (Fig. 2). A cosyntropin stimulation test (CST) performed following administration of 250 mcg of cosyntropin showed a baseline (at 4 Am) cortisol level of 0.95 µg/dL (26.6 nmol/L), with subsequent cortisol levels of 4.24 µg/dL (118.72 nmol/L) at 30 minutes and 4.95 µg/dL (138.6 nmol/L) at 60 minutes postinfusion (normal stimulation is >12.5 µg/dL; > 345 nmol/L in our assay, Roche). These results were consistent with cure of the AHC, with the expected postoperative hypocortisolism, and thus the patient started on a low, physiological dose of hydrocortisone.

## Outcome and Follow-up

The recovery was uneventful, with no adverse events reported. Given the excellent biochemical and clinical response to unilateral right adrenalectomy, further surgery, such as function-preserving (partial) left MBSA, has been deferred. Such therapy would only be pursued if she develops recurrent AHC from autonomous excessive cortisol secretion from the left adrenal gland, in situ. Within 3 months postsurgery, her features of MACS improved significantly, with weight reduction (body mass index 34.2 kg/m²) and resolution of bruising. The postoperative normalization of the biochemical indices after surgery (up to 15 months follow-up) are outlined in Table 1, with an increase in both ACTH and DHEA-S and improvement in the stimulated cortisol levels after CST, while undergoing gradual tapering of the glucocorticoid replacement dose. To confirm persistent nonclassical CAH after MACS resolution, an ACTH stimulation test was performed about 10 months after MBSA while on 7.5 mg hydrocortisone daily. Baseline 17-hydroxyprogesterone was 944 ng/dL (28.53 nmol/L), rising to 7730 ng/dL (233.5 nmol/L) at 60 minutes (confirmed by dilution; Labcorp Esoterix), diagnostic of nonclassical 21-hydroxylase deficiency.

## Discussion

Patients with 21-hydroxylase deficiency CAH, typically managed with glucocorticoids in symptomatic individuals, rarely develop endogenous hypercortisolism, making this case of recent adenoma development and MACS particularly noteworthy [1, 2]. The patient's initial CAH diagnosis, established 50 years ago, was characterized by hyperandrogenism, managed with long-term glucocorticoid therapy [1, 6]. This treatment, however, led to iatrogenic Cushing syndrome, a well-documented complication [3, 7, 8], prompting discontinuation after 20 years. It should be mentioned that a goal of glucocorticoid therapy is to use the lowest possible dose of hydrocortisone to provide physiologic cortisol levels while treating the symptoms of the disease—in her case, acne, hirsutism, and menstrual irregularities. During this time, the patient lived in a part of the world with poor access to expert endocrinology care, which likely contributed to overtreatment. After discontinuing glucocorticoid therapy, the iatrogenic hypercortisolism was resolved. Her subsequent stable eucortisolemia for 2 decades contrasts with the recent emergence of MACS. The biochemical diagnosis of MACS, evidenced by preoperative ACTH-independent hypercortisolism with elevated post-DST cortisol values, suppressed ACTH, and low DHEA-S, combined with profound postoperative hypocortisolism demonstrated by CST results, unequivocally confirms AHC and its surgical cure. Others have shown and we have personal experience with >2000 postadrenalectomy CSTs, and such a cortisol profile can only occur when the excised adrenal lesion had been secreting cortisol autonomously [9-11]. In normal physiological conditions or nonfunctional adenomas, cysts, unilateral pheochromocytomas, or other noncortisol-producing adrenal pathologies, the contralateral gland provides sufficient reserve to achieve normal cortisol levels poststimulation. The immediate postoperative hypocortisolism therefore constitutes definitive biochemical proof of cure of cortisol hypersecretion. This conclusion is further supported by the subsequent normalization of ACTH and DHEA-S levels and normalization of cortisol on follow-up. Sahlander et al's population-based study found a 109-fold higher CAH prevalence among individuals with adrenal tumors (20 of 26 573 cases) compared to controls, with many diagnoses following tumor detection, suggesting that chronic ACTH overstimulation from early CAH may predispose to later adrenal pathology [5]. The molecular driver of the adenoma causing MACS may involve somatic mutations, such as in *CTNNB1* (β-catenin), which could disrupt cellular regulation and promote autonomous cortisol secretion [12, 13]. Even in the absence of CAH, the management of MACS is not uncontroversial, and recommendations range from surveillance to surgery, where surgery seems to be associated with improvements in life expectancy and quality of life [14, 15].

The absence of genetic data, a consequence of the diagnosis era and the patient’s international residence, represents a key limitation. Nonetheless, the baseline and ACTH-stimulated 17-hydroxyprogesterone levels clearly confirm the underlying nonclassical CAH diagnosis after MACS resolution by adrenalectomy. Another limitation is the relatively short (15 months) surveillance period after MBSA to assess the risk of recurrent MACS in the contralateral gland. Comparative cases, such as those with unilateral adenomas and adrenal myelolipomas in classic CAH or mimicking nonclassical presentations, further highlight the variability in adrenal tumor development [16-18]. Long-term outcomes in CAH patients postadrenalectomy underscore the risk of adrenal crisis, yet this patient's recovery was uneventful, reflecting effective MBSA. Clinical guidelines recommend monitoring for adrenal incidentalomas in CAH, and this case reinforces the need for lifelong surveillance, especially given the potential for late-onset complications. Future research should explore genetic triggers and optimal screening protocols to prevent such rare sequelae.

## Learning Points

- People with longstanding CAH may develop MACS due to adrenal adenomas, requiring ongoing adrenal monitoring.
- Diagnosing MACS in people with CAH necessitates a thorough biochemical assessment after glucocorticoid cessation.
- MBSA offers a safe, effective treatment for MACS in this population.

## Contributors

All authors made individual contributions to authorship. R.A.M., M.L., A.K., and T.C. were responsible for data collection, analysis, and diagnosis and management of the patient as well as manuscript preparation. R.A.M. and T.C. were involved in the manuscript submission. All authors reviewed and approved the final draft.

## Data Availability

Data sharing is not applicable to this article as no datasets were generated or analyzed during the current study.
